# Safe patient handling: Exploring work constraints faced by healthcare workers in a Portuguese hospital

**DOI:** 10.1177/10519815261434531

**Published:** 2026-04-02

**Authors:** Joana Afonso-Fernandes, Marta Mendes, Pedro Arezes, Matilde A Rodrigues

**Affiliations:** 1 ALGORITMI Center, School of Engineering, University of Minho, 4700-058, Guimarães, Portugal; 2 RISE-Health, Center for Translational Health and Medical Biotechnology Research (TBIO), E2S, Polytechnic of Porto, 4200-072, Porto, Portugal; 3 Department of Safety at Work and Risk Management, Portuguese Oncology Institute of Porto (IPO Porto), 4200-072, Porto, Portugal

**Keywords:** cumulative trauma disorders, ergonomics, nursing staff, occupational injuries, risk management, work environment

## Abstract

**Background:**

Work-Related Musculoskeletal Disorders (WMSDs) remain a major occupational health concern in hospital settings, largely due to manual patient handling. Understanding the challenges faced by nurses and nursing assistants in patient handling is crucial for the effective implementation of safety interventions and the long-term protection of healthcare workers.

**Objective:**

This study aims to explore the work constraints experienced by nurses and nursing assistants during patient handling practices in a Portuguese hospital.

**Methods:**

A qualitative research design was adopted, combining 38 semi-structured interviews with non-participant observations.

**Results:**

Key constraints included staff shortages, deficiencies in care coordination, time constraints, and cultural resistance to change among nursing staff. Limited availability and inaccessibility of patient handling equipment were also frequently reported.

**Conclusions:**

The study proposes context-specific, data-driven recommendations to improve patient handling practices and promote a safer working environment for healthcare workers.

## Introduction

Work-Related Musculoskeletal Disorders (WMSDs) represent a significant global public health concern. According to Eurostat,^1^ they are among the most prevalent work-related health problems, with high rates in the health and social care sector. Healthcare workers are routinely exposed to multiple risk factors, among which manual patient handling is frequently identified as the most important.^2,3^ This involves lifting, transferring, and repositioning patients. These tasks are often performed manually and without assistive devices.^4^ Nurses and nursing assistants are particularly exposed, as these tasks are central to their daily routines.^5,6^ Several studies report higher rates of WMSDs among these workers compared to other healthcare occupations.^7,8^

WMSDs compromise workers’ health and quality of life, reduce performance, and increase absenteeism, as well as costs for healthcare institutions.^9–11^ In response, various interventions have been developed to protect healthcare workers from injury and improve patient safety during handling practices.^8,12^ Wåhlin et al.^13^ highlighted that the effectiveness of these interventions depends on employer commitment, the availability of appropriate equipment, and adequate training for healthcare professionals. The most widely adopted strategy to reduce physical workload during patient handling tasks is the use of mechanical lifting equipment and other assistive devices.^14,15^ These devices constitute the core components of Safe Patient Handling and Mobility (SPHM) programs, which aim to reduce the risk of injury.^16,17^

Despite these efforts, several barriers continue to hinder safe patient handling.^4,12,18^ These include limited equipment availability, insufficient training, worker-related factors, management commitment, and patient-related factors.^4,19,20^

Although WMSDs are highly prevalent among hospital nurses in Portugal,^7,21^ research specifically addressing patient handling practices remains limited.^22^ A better understanding of these practices is essential for the development of effective interventions and the safeguarding of healthcare workers’ health and safety. This study aims to explore the work constraints experienced by nurses and nursing assistants during patient handling tasks in a Portuguese public hospital, providing insights into the inconsistent implementation of safety measures.

## Methodology

### Study design

The study was conducted in a Portuguese public hospital, a healthcare institution with a triple mission: patient care, education, and research. The hospital is organized into specialized areas comprising clinics and services that provide personalized treatment tailored to patients’ needs. The hospital was selected based on three criteria: (i) access to and availability of willing participants; (ii) presence of improvement initiatives aligned with the study's objective; and (iii) hospital interest in enhancing patient handling practices.

Data on the constraints faced by healthcare workers during patient handling were collected from two main sources: semi-structured interviews and non-participant observations.

The study was reviewed and approved by both the hospital's Ethics Committee and Data Protection Officer.

### Sample

Participants were randomly selected during visits to the hospital's surgical inpatient units. Each was approached individually, provided with an overview of the study, and invited to participate voluntarily. The sample included 21 nurses (2 males, 9.5%; 19 females, 90.5%) and 17 nursing assistants (all females, 100%). Regarding professional experience, 7 participants had less than 10 years (18.4%), 20 had 10–20 years (52.6%), and 11 had 21 years or more (28.9%). Identification codes were assigned sequentially based on the order of the interviews. Participant characteristics are presented in Table 1.

**Table 1.** Sample description.

Category	ID	Gender	Experience in years
Nurses (n = 21)	N1	F	20
	N2	F	12
	N5	M	2
	N6	F	15
	N7	F	15
	N8	F	30
	N11	F	13
	N12	F	15
	N13	F	20
	N14	F	15
	N15	F	2
	N24	F	25
	N25	F	20
	N26	F	17
	N30	F	15
	N31	F	29
	N32	F	18
	N33	M	15
	N36	F	32
	N37	F	26
	N38	F	24
Nursing assistants (n = 17)	A3	F	6
	A4	F	30
	A9	F	7
	A10	F	5
	A16	F	16
	A17	F	42
	A18	F	22
	A19	F	10
	A20	F	4
	A21	F	13
	A22	F	12
	A23	F	17
	A27	F	19
	A28	F	22
	A29	F	4
	A34	F	22
	A35	F	19

Note: N = Nurse; A = Nursing Assistant; M = Male; F = Female.

### Semi-structured interviews

All interviews were conducted in person to capture a realistic, context-specific understanding of patient handling practices. Two semi-structured interview guides were developed, one for nurses (see Appendix A) and one for nursing assistants (see Appendix B). The interview questions were carefully designed to explore how patient handling tasks are performed in daily practice. Key topics were selected based on a review of the literature on WMSDs and the study's objectives, ensuring that the questions addressed relevant aspects of daily routines, decision-making processes, and the use of assistive devices. Open-ended questions were formulated to encourage participants to describe their experiences in detail. Both guides covered patient dependency assessment and communication, decision-making in patient handling, use of assistive devices, staff training and knowledge, and workload and staffing issues, with content tailored to the specific roles and responsibilities of each professional group.

Manual patient handling was defined as any patient handling task performed without assistive devices. Participants provided written informed consent after receiving both written and oral information about the study, including the voluntary nature of participation, the option to withdraw at any time and assurances of strict confidentiality. Only the research team had access to the original interview recordings, transcripts, and consent forms.

With the participants’ consent, all interviews were audio-recorded using a digital device to ensure accurate data collection. Interviews lasted approximately 20 min each. Data saturation guided the determination of the sample size.^23^ After 38 interviews, a rich dataset was obtained, as responses converged and no new significant insights emerged.

A thematic analysis was conducted on the interview transcripts to identify patterns and themes. Thematic analysis is a qualitative method for identifying themes.^24^ Following Javadi and Zarea,^25^ an inductive approach was used, with findings grounded entirely in the empirical data. Transcripts were read carefully multiple times to achieve deep familiarity with the data. Initial codes were generated from key concepts and significant responses. Similar or overlapping codes were iteratively refined and merged, then systematically organized into coherent subthemes and overarching themes. Relevant excerpts were grouped under each subtheme to accurately represent the data. This process ensured that the themes reflected patterns emerging from participants’ accounts.

### Non-participant observations

In addition to the interviews, observations of nurses and nursing assistants were conducted to gain a deeper understanding of the constraints reported during the interviews. The observations were unstructured, as recommended for qualitative, context-driven studies, allowing the researcher to draw on their own knowledge while refraining from active participation.^26^ They focused specifically on patient handling practices, including the execution of mobilization tasks, communication and coordination among healthcare workers, and the availability, accessibility, storage, and use of assistive devices within the surgical inpatient units.

Observations were conducted with the guidance of a nurse, who provided relevant contextual explanations to aid in the interpretation of observed situations. Field notes were recorded on the same day or the following day after each hospital visit to capture additional information that could complement and enrich the interview findings. Data collection concluded once sufficient information had been obtained to address the study's objectives.^27^ In total, approximately 14 h of observation were completed.

## Results

The qualitative analysis identified four major themes: organizational factors, equipment-related factors, worker-related factors, and patient-related factors. Table 2 summarizes the main themes and subthemes, showing the proportion of nurses (n = 21) who mentioned each subtheme. Figure 1 illustrates the frequency of mentions across all subthemes. Representative excerpts for each subtheme are presented below.

**Figure 1.**
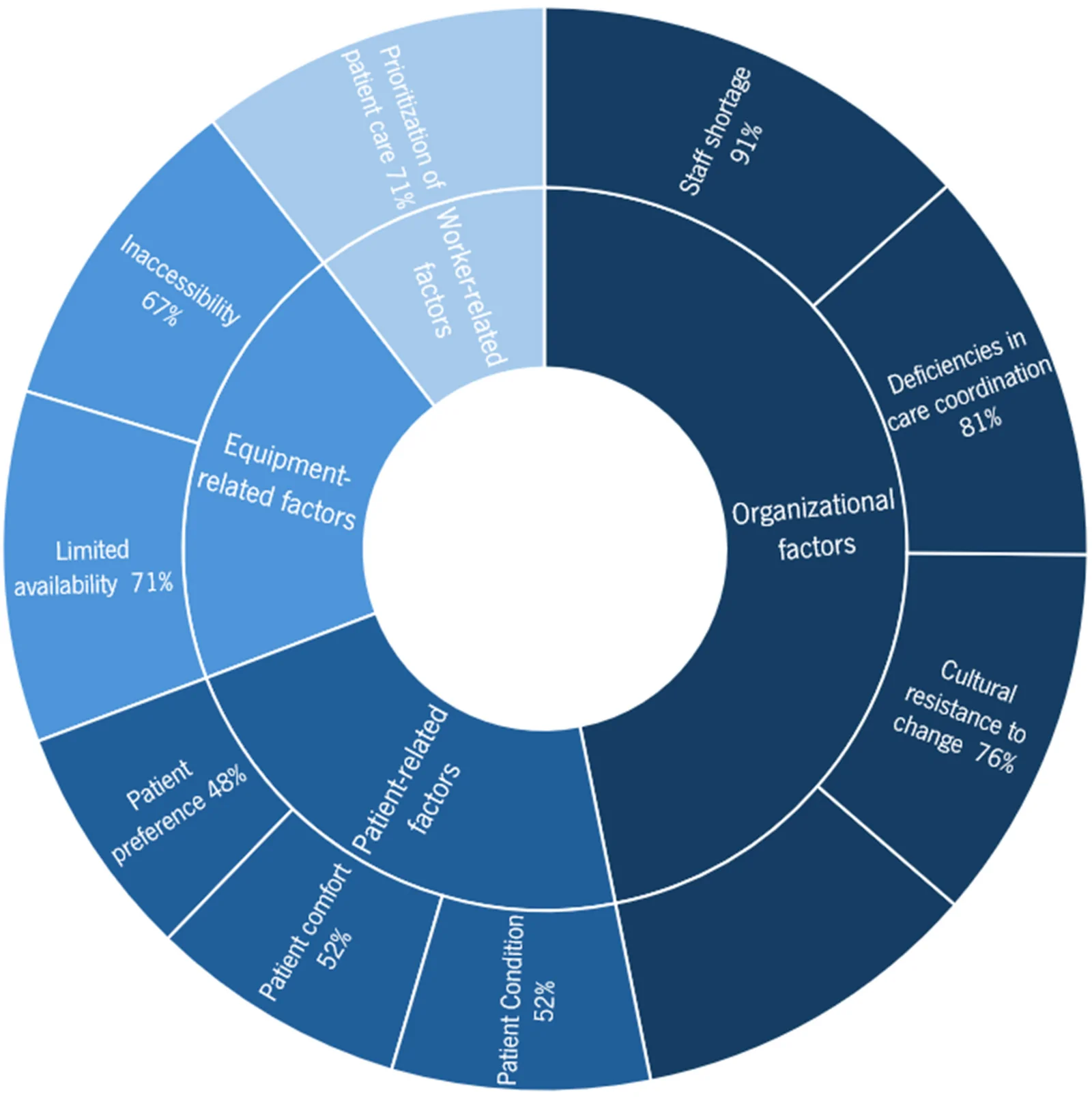
Frequency of subthemes mentioned by nurses.

**Table 2.** Themes and subthemes.

Main theme	subthemes	%
Organizational factors	Staff shortage	90.5
	Deficiencies in care coordination	80.9
	Cultural resistance to change	76.2
	Time constrainers	71.4
Equipment-related factors	Limited availability	71.4
	Inaccessibility	66.6
Worker-related factors	Prioritization of patient care	71.4
Patient-related factors	Patient comfort	52.4
	Patient Condition	52.4
	Patient preference	47.6

Organizational factors were the most frequently mentioned barriers to safe patient handling. This was primarily attributed to staff shortages, reported by 90.5% of participants. Participants consistently highlighted inadequate staffing levels, particularly during evening and night shifts, as well as on weekends, highlighting the need for additional team support during these periods.

“On the weekend, the number of patients is the same, the level of dependency is the same (…) and there are always fewer nurses, in every weekend shift.” [Nurse 12]

"Especially on weekends and nights, I don't agree with the reduction in staffing ratios because the care required is the same or even greater.” [Nurse 7]

Absenteeism was frequently highlighted as an aggravating factor due to the lack of effective mechanisms to ensure the timely replacement of absent staff.

“What happens is that, due to absenteeism and the specific characteristics of the service, many times the number of staff scheduled for a shift ends up not being fully met.” [Nurse 26]

“It only takes one nurse or one assistant to be absent and everything falls apart, because we are always operating at the bare minimum. Always.” [Nurse 14]

Another organizational subtheme was deficiencies in care coordination (80.9%), which emerged as a key factor in understanding decision-making related to patient handling. Nurses highlighted the lack of updated information regarding patients’ conditions prior to admission as a significant constraint.

"When the patient arrives at the ward, we are the ones who carry out the assessment, and it often does not match the level of dependency recorded in the patient profile.” [Nurse 7]

“We only become aware of the patient's level of dependency when patient arrive on the ward.” [Nurse 38]

Due to inconsistencies in the availability and accuracy of pre-existing information, nurses reported that they always conduct a mobility assessment upon patient admission. The information is recorded on a patient profile that is accessible to all staff. Although nurses stated that mobility assessments are performed before any intervention, they often lack sufficient time to update patient records due to workload.

"We don't always manage to update [the records]. There are shifts when we don't update them, so it carries over to the next day, and the patient, for example, is recorded as independent when he is not.” [Nurse 14]

Cultural resistance to change emerged as a relevant subtheme, reported by 76.2% of participants, particularly regarding the limited adoption of assistive devices in daily practice. Despite training and awareness of the importance of assistive devices and safe handling practices, their consistent integration into routine care remains limited. Participants emphasized that, despite knowledge and training, actual practices often diverge from theoretical guidelines.

“I think it's really a personal matter, a personal reluctance to use assistive devices. They [nurses] think it will make things harder instead of easier.” [Nurse 2]

“As much as we advocate for the use of assistive devices, and although people are increasingly aware of their existence, it is still something that requires a lot of effort and persistence. Only over time will it truly become part of the routine.” [Nurse 37]

“(…) as much as we are aware (…) we all have that information, training is mandatory, and we know that what happens is not that. Even during the training sessions, we share a lot of these experiences, that in theory things are one way, but in practice, it's not quite like that.” [Nurse 34]

In addition to cultural resistance to the adoption of assistive devices, several equipment-related factors were identified as barriers to their effective use. These primarily included limited availability (71.4%) and inaccessibility (66.6%) of the equipment.

“We should have more equipment available so we could use it without having to check if it's already been disinfected, if there's one available in the unit… having to go look for it…” [Nurse 14]

“If we request a lift equipment, it takes a long time. It's just not feasible.” [Nurse 32]

Difficulty in locating equipment was considered a significant barrier, particularly for lift devices, which were often unavailable within the unit. Time was also identified as a critical factor influencing nurses’ decisions to use assistive devices, as the process requires preparing the patient, locating the equipment and performing the mobilization. The unavailability of equipment during mobilization, combined with the time required to locate it, often leads nurses to perform patient handling manually.

"(…) we ask for help from colleagues because getting the lift equipment takes too long, and we don’t have that time (…).” [Nurse 10]

Patient- and worker-related factors were the least frequently mentioned in the interviews. Nurses consistently reported that attending to patients’ immediate needs often takes precedence, often resulting in patient handling being performed without the use of assistive devices.

“We prioritize the patient and patient care.” [Nurse 2]

"(…) there are few resources available, and between requesting them [assistive devices] and waiting for them to arrive, we’ve already lifted the patient because the priority is the patient and their needs. [Nurse 34]

The two most commonly reported patient-related factors were patient comfort (52.4%) and patient condition (52.4%). Nurses indicated that the discomfort associated with certain assistive devices often led to their non-use. On the other hand, the patient's condition sometimes prevented the use of these devices, necessitating manual mobilization. In more complex situations, such as handling heavier patients, nurses reported seeking assistance from colleagues.

“(…) well, we have assistive devices but they are uncomfortable for the patients. [Nurse 6]

“I prefer to use a sheet rather than the transfer device, which is always cold and rigid. It's not comfortable for the patient either.” [Nurse 26]

“Sometimes they're not practical [assistive devices], the patient has dressings or stitches, and it just doesn't work well. It becomes impractical.” [Nurse 36]

Additionally, nurses indicated that patient preference also influences the decision to use assistive equipment (47.6%). Several nurses reported that patients often preferred manual lifting over the use of mechanical assistive devices.

“When the patient knows that he is going to be lifted with the equipment, he immediately says that he does not want it.” [Nurse 37]

In addition to nurses, interviews were conducted with nursing assistants. Nursing assistants reported receiving information at the start of each shift about their assigned patients, however, this information was limited to patient identification. They do not handle patients independently, as decisions regarding patient handling are made by the nurse. Instructions on handling tasks and techniques are communicated verbally, typically by the nurse in charge of the patient. Nevertheless, nursing assistants mentioned that they had participated in training sessions offered by the hospital, where they learned various patient handling techniques and had the opportunity to practice using different assistive devices.

"The nurse tells us how we are going to handle that patient. We don't mobilize patients on our own, it's always with the nurse.” [Nursing assistant 16]

“We are informed about the patients assigned to us at the beginning of the shift. Normally, I go directly to the nurse assigned to that patient and ask for the details.” [Nursing assistant 30]

Regarding constraints on patient handling, nursing assistants highlighted staff shortages as a major issue, reported by 88.2% of participants.

“Many nursing assistants are absent. At the moment, we are working back-to-back shifts due to colleagues being on sick leave, others on vacation, and we have a limited number of staff. [Nursing Assistant 28]

“To do everything as outlined in the training, we need more staff (…).” [Nursing assistant 4]

## Discussion

Organizational factors emerged as the primary constraints, with staff shortages frequently reported by both nurses and nursing assistants. Similarly, Noble and Sweeney^20^ identified inadequate staffing levels as the most common barrier to the use of assistive devices during patient handling. Limited staffing and time pressures often discourage device use when it is perceived as more time-consuming than manual handling.^19^ Lee et al.^28^ further highlighted that insufficient staffing complicates access to immediate assistance when patients require multiple staff or additional equipment. Adequate staffing levels are therefore a critical resource for ensuring safe patient handling practices.^20,28^

Organizational constraints extend beyond staffing shortages to include deficiencies in care coordination. Significant gaps were identified in the availability and accuracy of patient information at admission. These gaps hinder the anticipation and allocation of appropriate handling resources, such as mechanical devices or additional staff, at a critical stage for risk mitigation. In addition, nurses often lack sufficient time to update patient records, which further compromises effective planning.

Cultural resistance to change was another commonly reported organizational factor. This factor has been consistently identified in previous studies as a barrier to the adoption of assistive devices.^29–31^ Fostering a culture of safety is essential for overcoming this resistance.^32^ Moreover, a positive safety climate has been shown to support safe patient handling practices among nurses.^33,34^

Although equipment-related factors are inherently organizational, they were examined separately in this study due to their direct impact on patient handling safety. Some assistive devices were available and, from a risk management perspective, had the potential to reduce exposure to musculoskeletal injury. However, their integration into daily practice was inconsistent.

Limited availability and inaccessibility were the most frequently reported barriers, consistent with previous findings.^20,29,34^ Despite being widely recognized as hazardous, whether performed by one or two workers,^34^ manual patient handling remains the predominant approach. This underscores the need to assess existing resources and invest in expanding equipment availability.

For example, the hospital had only two patient lifts shared across multiple units. This limitation helps explain the low frequency of equipment use reported by staff. Previous studies have demonstrated a strong association between greater equipment availability and increased utilization.^34,35^

Worker- and patient-related factors were reported less frequently than organizational constraints. Nurses indicated that, in order to meet patients’ immediate needs, they sometimes forgo the use of assistive equipment, particularly when it is perceived as time-consuming. This reflects an inherent tension between patient-centered care and occupational health and safety. Clinical demands and time pressures create complex trade-offs between competing priorities. Such pressures limit workers’ ability to follow prescribed procedures and require constant adaptation.^36^

Patient-related factors also played a significant role in nurses’ decision-making regarding the use of assistive devices. Our findings are consistent with previous research identifying key barriers, including patients’ comfort,^19,37^ clinical condition^29,37,38^ and individual preferences.^38,39^

Based on data from interviews and non-participant observations, a set of recommendations was developed to address the constraints identified by participants. These recommendations were grounded in the four major themes derived from thematic analysis. Each set of recommendations corresponds to the issues highlighted within its respective thematic domain. Table 3 summarizes these data-driven recommendations and illustrates how the qualitative findings informed actionable guidance for enhancing patient handling practices.

Despite the relevance of the proposed recommendations, successful implementation requires strong institutional commitment, adequate resource allocation, and sustained efforts to integrate safer patient handling practices into routine care. Without such long-term investment, these improvements are unlikely to be maintained.

In Portugal, the general legal framework for occupational safety and health (Law no. 102/2009) requires employers, including hospitals, to adopt preventive measures to eliminate or minimize occupational risks. However, no specific legislation currently mandates the implementation of structured SPHM programs in healthcare settings. Although the law establishes a general duty to ensure worker safety, the absence of targeted regulation may hinder the consistent adoption of evidence-based practices.

Implementing an SPHM program is essential for promoting safer practices and reducing musculoskeletal injury risks among healthcare workers. Previous studies have shown that such programs effectively reduce musculoskeletal injury rates and offer economic advantages.^16,17,40^ Mandating SPHM programs through national legislation, as in the United States of America (USA), the United Kingdom, and Australia, could further enhance compliance. In the USA, several states legally require SPHM programs in hospitals, and healthcare workers in these states report more consistent use of assistive devices and safer handling practices.^41^ For example, the use of mechanical lifting equipment is significantly higher in states with relevant legislation, suggesting a positive influence on practice.^41^ Lee et al.^42^ also found that California's SPHM legislation significantly improved hospital policies, programs, and workers’ musculoskeletal health. Nevertheless, successful implementation may depend on the specific characteristics of individual hospitals.

Although this study provides valuable insights, several limitations should be acknowledged. First, the study was conducted in a single hospital, which limits the generalizability of the findings. Second, the semi-structured interviews did not explicitly distinguish between mechanical and non-mechanical assistive devices, potentially affecting response accuracy. Third, the researcher's presence during in situ observations may have introduced a Hawthorne effect, influencing participants’ behavior. In addition, the exclusive reliance on qualitative data, without complementary quantitative assessment, further restricts generalizability. Ambiguities in interview phrasing and terminology may also have introduced interpretive variability. Future research should address these limitations by including multiple Portuguese hospitals, clearly differentiating between types of assistive devices, and adopting mixed methods approaches.

**Table 3.** Recommendations for increasing safe patient handling practices**.**

Domain	Recommendation
Planning and Assessment	- Ensure that staffing ratios account for the time required for patient handling assessments. - Establish written care plans to ensure consistent transfer of information regarding patient handling needs. - Assess and determine the most appropriate assistive equipment for each hospital unit, considering the specific needs of the patient population. - Implement decision-making algorithms to support the identification of appropriate equipment and staffing requirements.
Equipment	- Ensure that equipment is stored in easily accessible locations.
Training	- Staff competencies should be regularly reassessed and reinforced through ongoing training.
Engagement and Communication	- Involve staff in decision-making processes to promote engagement and ensure the practical applicability of interventions. - Facilitate mechanisms that enable continuous learning from actual work practices.
Patient Involvement	- Demonstrate the use of assistive equipment to patients. - Provide patients with clear information about the use of assistive devices to promote cooperation.

## Conclusions

This study identified the main constraints influencing patient handling practices in a Portuguese hospital. Key challenges included organizational factors such as staff shortages, deficiencies in care coordination, time constraints, and cultural resistance to change among nursing staff. Despite some measures to reduce patient handling risks, ongoing efforts are needed to ensure the proper use of assistive devices and adherence to safety practices. Practical recommendations were proposed, but their success depends on sustained investment and strong institutional support. Mandating SPHM programs through national legislation could further enhance safety and efficiency.

## Appendix A – Interview Guide for Nurses

1. When a new patient is admitted to the unit, is there any information available regarding their level of dependency, and how do you use it in your work?
2. How do you communicate to the nursing assistant the patient's required self-care and level of dependency? Please provide examples.
3. For patients with a high level of dependency, how many professionals are involved in their handling and which professionals?
4. Which patient handling technique do you use most frequently during your shift and why?
5. What factors do you consider when determining the most appropriate handling technique?
6. How do you prepare to handle a patient?
7. Do you have the necessary knowledge to perform the task? If so, how was this knowledge acquired?
8. Are assistive devices available in the unit? How does their availability affect your work?
9. Are these assistive devices generally in place and sufficient? Please explain.
10. Do you have enough time to safely handle the patient, or do you sometimes need to rush? Please provide some examples of when this occurs.
11. Have you ever had to adjust the way you handle a patient? How and why?
12. Does the number of assigned patients affect the time pressure associated with patient handling? How do you cope with it?
13. How do you perceive the adequacy of the number of nurses per shift?
14. How do you perceive the adequacy of the number of nursing assistants per shift?
15. How do you proceed when there is a staff shortage (e.g., illness, vacation)?

## Appendix B – Interview Guide for Nursing Assistants

1. How are you informed about the patient handling tasks to be performed?
2. How do you determine which handling technique should be used for each patient?
3. Does the timing of receiving information about a patient's condition affect the time pressure during patient handling? If so, how?
4. What types of handling activities do you perform alone?
5. How do you prepare to handle a patient?
6. Do you have the necessary knowledge to carry out handling tasks? If so, how was it acquired?
7. Do you have enough time to safely perform patient handling, or do you sometimes need to rush? Please provide some examples of when this occurs.
8. Are assistive devices available in the unit to facilitate patient handling? In what ways does this affect your work?
9. Are these assistive devices generally in place and sufficient? Please explain.
10. How do you perceive the adequacy of the number of nurses per shift?
11. How do you perceive the adequacy of the number of nursing assistants per shift?
12. How do you proceed when there is a staff shortage (e.g., illness, vacation)?
